# Pain Relief in Neonates

**Published:** 2013-04-01

**Authors:** Lalitha Krishnan

**Affiliations:** Department of Pediatrics, Pondicherry Institute of Medical sciences

 It seems unbelievable how long it took the medical community to realize that newborns also feel pain.

It is the basic right of every individual, irrespective of age or size, to have alleviation of pain. Pain in newborn infants is a ubiquitous phenomenon. All newborns, even normal ones, will experience iatrogenic pain in the first days of life, commencing with vitamin K injection and blood collection for sugars, bilirubin or metabolic screening before discharge from the hospital. Neonates admitted to present day neonatal intensive care units (NICU) are constantly exposed to pain, discomfort or noxious stimuli of variable intensity for a variety of reasons. These include major surgical procedures, needle pricks for blood drawing and cannulations. The painful situation may be short lived or chronic as in the case of necrotizing enterocolitis and prolonged ventilation. Even apparently innocuous care giving procedures like diaper changes, daily weighing and removal of adhesive tape results in noxious stimuli. All these events, especially in preterm infants individually or cumulatively, result in adverse sequelae in the form of death, poor neurologic outcomes, abnormal somatization and response to pain later in life. 

**Neonatal pain myths**

Evaluation of pain is considered difficult in neonates and young infants as pain has been considered a subjective phenomenon. Early studies of neurologic development concluded that neonatal responses to painful stimuli were decorticate in nature and that perception or localization of pain was not present. Furthermore, because neonates may not have memories of painful experiences, they were not thought capable of interpreting pain in a manner similar to that of adults. On a theoretical basis, it was also argued that a high threshold of painful stimuli may be adaptive in protecting infants from pain during birth. These traditional views have led to a widespread belief in the medical community that the human neonate or fetus may not be capable of perceiving pain [1].

**Definitions**

Pain: The International Association of the Study of Pain (IASP) [2] defines that pain is “an unpleasant sensory and emotional experience associated with actual or potential tissue damage or described in terms of such damage”. According to the IASP, pain is always subjective. Each individual learns the application of the word through experiences related to injury in early life” However, this definition of pain by the IASP does not apply to humans incapable of self-reporting pain e.g. newborn and older infants. Anand and coworkers [3] state that the “relationships between feeling pain and reporting pain are highly context-dependent “. Since the 1980’s it has become increasingly evident that the fetus and newborn perceives and responds to pain. If pain is prolonged or repetitive, the developing pain system may be modified permanently, resulting in altered processing at the spinal and supraspinal levels [4]. Over the last several years, evidence from both clinical and preclinical research has shown that newborns are more sensitive to pain than older infants, children, and adult. For healthy newborns painful experiences are limited to a heel prick or venepuncture for metabolic screening or intramuscular injection of vitamin K or vaccines. For preterm or ill term-neonates, the experience is very different. They are exposed to repeated procedural pain [5], extensive tissue damage resulting from surgery, or the invasiveness of endotracheal tubes placed for mechanical ventilation. Thus, at a time when most healthy term infants are learning about their environment and preterm infants are growing in the protective uterine environment, approximately 8% of neonates are coping with pain that, if left untreated, will interfere with normal growth and development [6]. Multiple sources of clinical and experimental evidence support the need for providing adequate analgesia/anesthesia for newborns who undergo invasive procedures (medical, surgical, diagnostic, and therapeutic) or develop conditions associated with a significant component of pain (eg, skin burns, necrotizing enterocolitis) [7]. 

Nociception: This is defined as the ability to feel pain caused by the stimulation of a nociceptor. Nociceptors are pain receptors in the somatic and visceral organs that can detect mechanical, thermal or chemical changes above a set threshold. Once stimulated, a nociceptor transmits a signal along the spinal cord to the brain. Nociception triggers a variety of autonomic responses and may also result in a subjective experience of pain in conscious beings. It comprises of four stages; transduction, transmission, modulation and perception.

In literature, terms relating to pain and nociception are used interchangeably and in this review the two will be considered the same. 

Development of nociception in the fetus and newborn: 

The neural pathways for nociception as shown above are traceable in the newborn and the density of pain fibres in the skin are similar to that of adults [8]. Electron microscopy and immunocytochemical studies show that the development of various types of cells in the dorsal horn (along with their laminar arrangement, synaptic interconnections, and specific neurotransmitter vesicles) begins before 13 to 14 weeks of gestation and is completed by 30 weeks [9]. Lack of myelination has been used as an index of immaturity and often cited as reason for neonates to be incapable of feeling pain. But even in the peripheral nerves of adults, nociceptive impulses are carried through unmyelinated (C-polymodal) and thinly myelinated (A-delta) fibers [10]. Moreover, pain pathways to the spinal cord, brain stem and thalamus are completely myelinated by 30 weeks; whereas the thalamo-cortical pain fibers in the posterior limb of the internal capsule and corona radiata are myelinated by 37 weeks [11]. Infants as young as 25 weeks post menstrual age (PMA) have been shown to have cortical responses to noxious stimuli [12, 13]. Near infrared spectroscopy studies in preterm infants from 28-36 weeks gestation undergoing tactile, non-noxious and painful stimuli (venepuncture) found that somatosensory cortical activation occurs bilaterally following unilateral stimulation. These suggest that neonates do have the required neuronal connections to experience the affective components of pain. 

**Pain neurotransmitters **

Various substances have been identified for transmission and control of pain but substance P is the one best investigated in babies in whom significant levels were demonstrated [14]. Endogenous opioids are released in the human fetus at birth and in response to fetal and neonatal distress [15].

**Changes during pain**

Physiological: 

Changes in heart rate, oxygenation and palmar sweating have been observed in neonates undergoing painful clinical procedures. The magnitude of changes in the heart rate was related to the intensity and duration of the stimulus and to the individual temperaments of the babies. Large fluctuations in oxygenation above and below an arbitrary "safe" range of 50 to 100 mm Hg have been observed during various surgical procedures in neonates. Tracheal intubation in awake preterm and full-term neonates caused significant hypoxemia together with increases in arterial blood pressure and intracranial pressure. The increases in intracranial pressure with intubation were abolished in preterm neonates who were anesthetized [16]. In addition, infants' cardiovascular responses to tracheal suctioning were abolished by opiate-induced analgesia [17].

Hormonal and Metabolic:

Plasma renin activity increased after venepuncture in full-term neonates. In preterm neonates receiving ventilation therapy, chest physiotherapy and endotracheal suctioning showed large increases in plasma epinephrine and norepinephrine; this response was decreased in sedated infants [18]. In neonates undergoing circumcision without anesthesia, plasma cortisol levels increased markedly during and after the procedure. Preterm and full-term neonates who underwent surgery under minimal anesthesia documented a marked release of catecholamines, growth hormone, glucagon, cortisol, aldosterone, and other corticosteroids, as well as suppression of insulin secretion. These results indicate that the nociceptive stimuli during surgery performed with minimal anesthesia were responsible for the massive stress responses of neonates. 

**Consequences of pain**

Medical:

Pain may worsen already compromised physiological states like hypoxia, hypercarbia, acidosis, hyperglycemia or respiratory distress. Babies who received good peri-operative analgesia showed stable course and faster recovery. 

Neurodevelopmental:

Preterm infants <1000g who have been exposed to repeated noxious stimuli are less responsive to painful stimuli at 18 months of age but at 10 years of age rate medical pain higher than their normal weight counterparts

General principles in the prevention and management of pain in newborns:

1. Neuroanatomical components and neuroendocrine systems are sufficiently developed to allow transmission of painful stimuli in the neonate.
2. Pain in newborns is often unrecognized and undertreated. Neonates do feel pain, and analgesia should be prescribed when indicated during medical care.
3. If a procedure is painful in adults it should be considered painful in newborns, even if they are preterm.
4. Compared with older age groups, newborns may experience a greater sensitivity to pain and are more susceptible to the long-term effects of painful stimulation.
5. Adequate treatment of pain may be associated with decreased clinical complications and decreased mortality.
6. Sedation does not provide pain relief and may mask the neonate's response to pain.
7. A lack of behavioural responses (including crying and movement) does not necessarily indicate a lack of pain.
8. Severity of pain and the effects of analgesia can be assessed in the neonate. Health care professionals have the responsibility for providing a systematic approach to pain management including assessment, prevention and treatment of pain in neonates.
9. Treatment should include the appropriate use of environmental, behavioural and pharmacological interventions.
10. Environment should be as conducive as possible to the well being of the neonate and family.
11. Education and validation of competency in pain assessment and management for all neonatal doctors and nurses, is a professional responsibility of clinical units.

Neonatal pain control: All neonatal units are required to have a neonatal pain control program which emphasizes the following [19].

1. Providing routine assessments to detect neonatal pain
2. Reducing the number of painful procedures
3. Preventing or treating acute pain from bedside invasive procedures
4. Anticipating and treating postoperative pain following surgery
5. Avoiding prolonged or repetitive pain and stress during neonatal intensive care

**Pain assessment scales: The fifth vital sign** [20]

Selecting the most appropriate tool for evaluating neonatal pain is essential to its management. Documentation of pain is also crucial as there can be variation in pain perception in babies between various caregivers. Many pain scoring tools exist and a few that are used commonly are given in Table 1.

**Figure:**
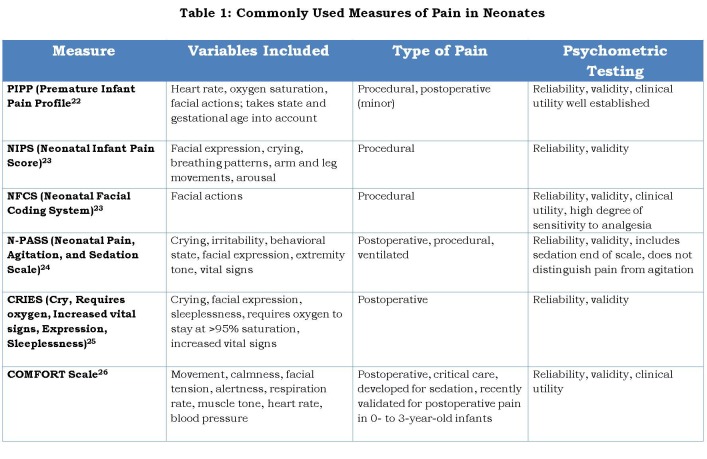
Table 1: Commonly Used Measures of Pain in Neonates

**Pain management in neonates**

Multiple classes of drugs have been evaluated for the prevention and management of neonatal pain and stress, including opioid analgesics, local anesthetics, general anesthetics, sedatives, hypnotics, non-steroidal anti-inflammatory drugs (NSAIDs), and sucrose (Table 2). Although much research has been performed with these agents, many questions remain unanswered thus preventing the optimal use of these drugs in clinical practice [27]. Pain in neonates can be managed by pharmacological and non-pharmacological interventions. Using analgesics to relieve short-term procedural pain in newborns is questionable because of these agents’ poor effectiveness and potential side effects [28]. Non-pharmacological pain relief strategies are convenient, inexpensive, can be used without prescriptions, and are also well tolerated by infants. Procedural pain in newborns has been relieved by non-pharmacological interventions, such as nonnutritive sucking (NNS) [29] swaddling, facilitated tucking [30], oral sucrose [31, 32], breast feeding [33] and skin-to-skin contact [34].

Behavioral approach:

Good planning will result in avoiding redundant and unnecessary blood sampling. Care should be taken to avoid other routine care before a prick. Baby should be well swaddled and preferably held by the mother. If situation allows, procedure should be done during or after a feed. Eyes should be shielded from the glare of procedure lamps. After the procedure baby should be held and comforted till all cues of pain have disappeared.

**Figure:**
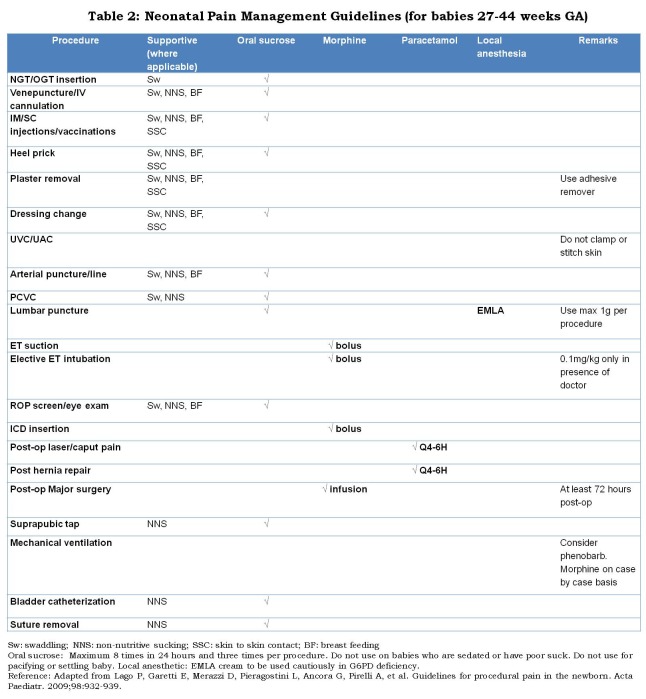
Table 2: Neonatal Pain Management Guidelines (for babies 27-44 weeks GA)

Procedural pain relief:

1. Non-nutritive sucking: [29] Using a pacifier would not be a feasible option in India because of the obvious disadvantages. Pacifiers are dipped into sucrose solutions and given to babies to combine the synergism of non-nutritive sucking with sucrose analgesia [35].
2. Breast feeding: Babies in a comfortable position in the mother’s arms and breast feeding showed a statistically significant difference in the duration of crying during and after immunization [33]. This has potential for use in well babies especially in immunization clinics.
3. Swaddling: or facilitative tucking of the infant ensures smooth execution of procedure but this is feasible only in certain infants and also depends on the procedure. Blood drawing from extremities would benefit by tucking [36].
4. Kangaroo care: Gray et al [37] found that 10–15 minutes of kangaroo care reduced crying, grimacing, and heart rate during heel-stick procedures. Johnston et al showed that kangaroo care significantly reduced the acute pain responses of preterm neonates at 32–36 weeks’ and 28–32 weeks gestation [38].
5. Oral Sucrose; Oral sucrose and other sweet tasting solutions have been used to promote calm and to reduce pain in infants over the past century, and even before this time. In 1991 Blass [39] reported that 2mL 12%sucrose compared with 2mL water significantly reduced crying time during heel prick and circumcision. The underlying mechanism of the analgesic effects of sweet tasting solutions is considered to be due to an orally mediated release of endogenous opioids. Calming effects were shown to be due to sweet taste, and not volume dependent, as small volumes of 0.2 mL sucrose were equally as effective as larger volumes of 0.6 mL and 1.0 mL. The effects of sweet taste peak at two minutes following administration, and persist for around five to eight minutes [40] and are dependent on contact with the tongue, and not sweet ingestion directly via a nasogastric tube [41]. Despite a large number of studies the mechanism of sweet taste and pain protection is unclear.

**Guidelines for using oral sucrose in neonates**

Indications for use: Any short-term procedural pain

1. Intravenous access
2. IM injection
3. Tape removal
4. Lumbar Puncture
5. Minor suturing
6. Arterial or venous blood sampling
7. Suctioning (i.e. nasal)
8. Urinary catheterization
9. Suprapubic tap
10. NG/OG insertion
11. Dressing change
12. Immunization
13. ROP exam
14. Chest tube insertion/removal

Principles:

1. 24% sucrose water when placed in the mouth, induces endogenous opioid production providing analgesia for minor procedures
2. Do not use more than 3 doses during a single procedure
3. Do not use for infants requiring ongoing pain relief (e.g. postoperative), since these infants will require acetaminophen or an opioid such as fentanyl or morphine.
4. It is important to realize that although an infant may still cry and show signs of pain when 24% sucrose water is used, studies have consistently shown that the sensation of pain and its negative effects will be diminished.
5. Analgesic effect of 24% sucrose water appears to be less effective after 46 weeks post conceptual age.

Dosages: ONLY oral administration/dose

1. Intubated infants: 0.1ml
2. Infants < 1000 grams: 0.1ml
3. Infants <= 28 week gestation: 0.1ml
4. Infants >= 1000 to 2000 grams: 0.1-0.2ml
5. Infants >= 2000 grams: 0.1-0.5ml

Procedure:

1. Using a 1ml sterile syringe or a dropper, draw up desired dose, place tip of syringe/dropper into the infant’s mouth onto anterior portion of the tongue and dispense solution slowly, allow the baby to savour the sweetness.
2. Wait 2 minutes and then perform intervention
3. For infants requiring occasional sucrose doses, nurse may draw dose directly from container (discarding when procedure is completed).
4. If giving more than 0.1ml, it may be best to give a portion of the dose 2 minutes prior to the procedure, and then the remainder of the dose intermittently, throughout the procedure.

Contraindications:

Use of 24% sucrose water is contraindicated in the following infants:

1. Infants at high risk for NEC; a. Asphyxiated infants, b. Infants with congenital heart disease that are not on established feeds, c. Infants with feeding intolerance, d. Infants without bowel sounds
2. Infants with esophageal atresia or tracheal esophageal fistula
3. Infants who are sedated or on other pain medications that are at risk for aspiration
4. Post-op infants who need to avoid excessive saliva production
5. Infants with active phase PPHN

Documentation:

1. Document on nursing flowsheet/medication area the amount and # of doses used.
2. Assess pain score using a suitable scale before, during, and after the procedure documenting on the nursing flowsheet.
3. Repeat doses may be administered during single procedure if indicated by pain score, not exceed 3 doses.

Concomitant use of various non-pharmacological techniques achieves greater clinical effectiveness than any one of these techniques used alone

**Local anesthetics**

Cutaneous infiltration of lidocaine or other local anesthetics treats pain from skin-breaking procedures like lumbar puncture, ICD insertion, for about 60-90 minutes [42]. EMLA cream (eutectic mixture of local anesthetic) has been used for circumcision but studies have shown that it is effective but inferior to dorsal penile nerve block [43]. Disadvantage includes the prolonged time for onset of action. For elective planned procedures e.g. lumbar puncture, circumcision, intravenous lines, arterial lines, where more than 60 minutes time is available, EMLA cream is helpful. Interestingly, EMLA cream is not useful in heel prick pain [44]. Anesthetic eye drops in combination with oral sucrose have been tried for reducing pain during retinopathy of prematurity (ROP) screening.

**Regional anesthesia**

This may be used appropriately e.g. dorsal penile block for circumcision if there is sufficient knowledge of techniques and dosages of various agents [45].

**Peri-operative pain relief**

Millions of newborns undergo surgery for various conditions around the world every year. Pain interventions must plan for intra-operative and post-operative periods. Potential drug therapeutic groups include opioids and opioid antagonists, sedatives/hypnotics, vapor anesthetics, local anesthetics, or NSAIDs, and there is opportunity to combine multiple types of analgesic intervention

**Opioid analgesics**

Morphine: This is useful for moderate to severe acute pain, for pre-operative sedation, and during anesthesia. Morphine and its metabolites are cleared by the kidneys and partly by biliary excretion. It is administered usually by a continuous infusion of 10-30µg/kg/hour in ventilated neonates for perioperative pain relief [46]. Neonates, especially preterms are more sensitive to opioids and are at risk for apnea, hypotension and urinary retention.

Fentanyl: This is a synthetic opioid that is 50-100 times more potent than morphine. The main side effects are apnea, bradycardia and chest wall rigidity. In ventilated neonates both morphine and fentanyl infusions produce evidence of physiological pain relief but may prolong ventilation [47].

Others: Remifentanil and alfentanyl have been used for short procedures like tracheal intubation or placement of central lines but safety data are lacking in neonates [48].

**Non-opioid analgesics**

Acetaminophen: (paracetamol) is often prescribed to manage mild to moderate procedural or post-operative pain. Data on newborn pain relief has been generally negative but it is effective in ages 3-6 months and older. Plasma clearance of acetaminophen is slower in neonates and hence should be administered in a dose of 10-15mg/kg orally or 20-25mg/kg rectally every 6-8 hours. 

**CONCLUSIONS**

Despite published data on the complex behavioral, physiologic, and biochemical responses of neonates and the detrimental short- and long-term clinical outcomes of exposure to repetitive pain, clinical use of pain-control measures in neonates undergoing invasive procedures remains sporadic and suboptimal.

## Footnotes

**Source of Support:** Nil

**Conflict of Interest:** None
